# Genome Sequences for Three Denitrifying Bacterial Strains Isolated from a Uranium- and Nitrate-Contaminated Subsurface Environment

**DOI:** 10.1128/genomeA.00449-13

**Published:** 2013-07-05

**Authors:** Raghavee Venkatramanan, Om Prakash, Tanja Woyke, Patrick Chain, Lynne A. Goodwin, David Watson, Scott Brooks, Joel E. Kostka, Stefan J. Green

**Affiliations:** Department of Bioengineering, University of Illinois at Chicago, Chicago, Illinois, USAa; National Centre for Cell Science, Pune, Maharashtra, Indiab; Joint Genome Institute, Walnut Creek, California, USAc; Los Alamos National Laboratory, Los Alamos, New Mexico, USAd; Environmental Sciences Division, Oak Ridge National Laboratory, Oak Ridge, Tennessee, USAe; Schools of Biology & Earth and Atmospheric Science, Georgia Institute of Technology, Atlanta, Georgia, USAf; DNA Services Facility, University of Illinois at Chicago, Chicago, Illinois, USAg; Department of Biological Sciences, University of Illinois at Chicago, Chicago, Illinois, USAh

## Abstract

Genome sequences for three strains of denitrifying bacteria (*Alphaproteobacteria*—*Afipia* sp. strain 1NLS2 and *Hyphomicrobium denitrificans* strain 1NES1; *Firmicutes*—*Bacillus* sp. strain 1NLA3E) isolated from the nitrate- and uranium-contaminated subsurface of the Oak Ridge Integrated Field Research Challenge (ORIFRC) site, Oak Ridge Reservation, TN, are reported.

## GENOME ANNOUNCEMENT

Nitrate is a key groundwater contaminant at nuclear legacy sites such as the Oak Ridge Integrated Field Research Challenge (ORIFRC) site, Oak Ridge Reservation, TN, where nitrate concentrations can exceed hundreds of millimoles (1). To better understand the denitrification capacity of the bacterial community, we isolated bacteria from the site under denitrifying conditions (2). Bacteria from five genera were isolated (*Afipia*, *Hyphomicrobium*, *Rhodanobacter*, *Intrasporangium*, and *Bacillus*). Genome sequences from *Rhodanobacter* isolates have been described previously (3). Here, we present the complete or nearly complete genomes of three additional denitrifying bacteria isolated from the ORIFRC site (2).

Genomic DNA was extracted using the Bacterial CTAB Protocol provided by the Joint Genome Institute. Genome sequence data were generated using paired-end Illumina and Roche 454 mate-pair sequencing and manual finishing steps, essentially as described previously (e.g., reference 4). Sequence data were assembled using NEWBLER v.2.3, and the Phred/Phrap/Consed software was used for assembly and quality assessment in the finishing process. Annotation was performed using NCBI’s Prokaryotic Genomes Automatic Annotation Pipeline (PGAAP).

*Afipia* sp. strain 1NLS2 (with 99% small-subunit [SSU] rRNA gene sequence similarity to *A. felis* ATCC 53690 [accession no. AGWZ01000001]) was grown with at least 25 mM nitrate under denitrifying conditions, and it reduced nitrate to nitrogen gas. The draft genome is composed of 10 contigs totaling 3,971,756 bases (*N*_50_, 612,490 bases; maximum contig length, 1.78 Mb). GC content is 61.3%, and 3,876 putative genes were identified. A single rRNA operon and 50 tRNA genes were detected. Genes for complete denitrification to N_2_ were identified, including genes encoding nitric and nitrous oxide reductases. Nitrate and nitrite reductase genes were similar to those in strain ATCC 53690, but no nitric or nitrous oxide reductase genes were identified in ATCC 53690.

*Hyphomicrobium denitrificans* strain 1NES1 (with 99.7% SSU rRNA gene sequence similarity to *H. denitrificans* ATCC 51888 [accession no. CP002083]) reduced nitrate to nitrogen gas and did not accumulate nitrous oxide during growth under denitrifying conditions. Growth was markedly slower for *H. denitrificans* than for *Afipia* and *Bacillus* strains. The complete genome is composed of a single chromosome of 3,808,687 bases, with a GC content of 59.7% and 3,778 putative genes. A single rRNA operon and 47 tRNA genes were detected. Genes for complete denitrification to N_2_ were identified.

*Bacillus* sp. strain 1NLA3E (with 98% rRNA gene sequence similarity to *B. bataviensis* [accession no. AJLS01000166]) reduced nitrate to nitrous oxide; no nitrogen gas was produced. The complete genome is composed of a single chromosome of 4,815,602, with a GC content of 38.0% and 4,833 putative genes. Twelve rRNA operons and 88 tRNA genes were identified. Some denitrification genes were identified through automated annotation (e.g., nitrate reductase, periplasmic, large subunit, *napA*; nitric oxide reductase, *qnorB*). A gene for a putative copper-containing nitrite reductase was identified by comparative BLAST analysis but not through automated annotation (i.e., locus tag B1NLA3E_19505). Genes potentially involved in nitrous oxide reduction were identified (*nosL*, *nosD*), but no other nitrous oxide reductase genes were detected.

### Nucleotide sequence accession numbers.

The genome sequences were deposited in GenBank under the following accession numbers: *Bacillus* sp. strain 1NLA3E, CP005586; *Hyphomicrobium denitrificans* strain 1NES1, CP005587; and *Afipia* sp. strain 1NLS2, ADVZ00000000.
